# Using Single-Cell RNA Sequencing and MicroRNA Targeting Data to Improve Colorectal Cancer Survival Prediction

**DOI:** 10.3390/cells12020228

**Published:** 2023-01-05

**Authors:** Andrew Willems, Nicholas Panchy, Tian Hong

**Affiliations:** 1 School of Genome Science and Technology, The University of Tennessee, Knoxville, TN 37916, USA; 2 Institute for Cyber-Enabled Research, Michigan State University, East Lansing, MI 48824, USA; 3 Department of Biochemistry & Cellular and Molecular Biology, The University of Tennessee, Knoxville, TN 37996, USA; 4 National Institute for Mathematical and Biological Synthesis, Knoxville, TN 37996, USA

**Keywords:** colon cancer, microRNA, single-cell RNA-sequencing

## Abstract

Colorectal cancer has proven to be difficult to treat as it is the second leading cause of cancer death for both men and women worldwide. Recent work has shown the importance of microRNA (miRNA) in the progression and metastasis of colorectal cancer. Here, we develop a metric based on miRNA-gene target interactions, previously validated to be associated with colorectal cancer. We use this metric with a regularized Cox model to produce a small set of top-performing genes related to colon cancer. We show that using the miRNA metric and a Cox model led to a meaningful improvement in colon cancer survival prediction and correct patient risk stratification. We show that our approach outperforms existing methods and that the top genes identified by our process are implicated in NOTCH3 signaling and general metabolism pathways, which are essential to colon cancer progression.

## 1. Introduction

Colorectal cancer (CRC) is estimated to develop in 945,000 patients worldwide yearly, with approximately 492,000 dying from the disease. Its complexity leads to challenges with interpreting the relationship between the many inputs of classical statistical models and their outputs related to large datasets. Current work in predicting the prognosis of CRC, using gene expression data, has focused on identifying novel biomarkers to predict survival and treatment outcomes through differential gene expression [1,2]. These approaches have been developed to work with next-generation bulk RNA sequencing (RNA-seq) [3,4,5], a technique that only generates the averages of gene expression across cells. Models that prioritize marker genes, based on bulk RNA-seq, are, therefore, unable to describe and utilize widely observed cancer cell heterogeneity for prognostic predictions [6,7,8]. In addition to the limitation in data, many prognostic models for colorectal cancer use unregularized Cox models to perform survival analysis. This can lead to issues with overfitting to training data and challenges with including additional inputs to a model.

Controlling gene expression at the post-transcriptional level is not only crucial for cancer progression, but also potentially useful for developing anticancer therapeutics. MicroRNAs (miRNAs) are small (22 nts), post-transcriptional regulators of messenger RNA. They have been studied in various systems and have recently been found to play essential roles in cancer regulation and progression [9,10,11,12]. While there are a variety of approaches to identifying biomarkers across different cancers, it remains challenging to integrate miRNA target information with RNA-sequencing data, primarily containing mRNA transcripts for prognostic predictions of CRC.

**Figure 1:**
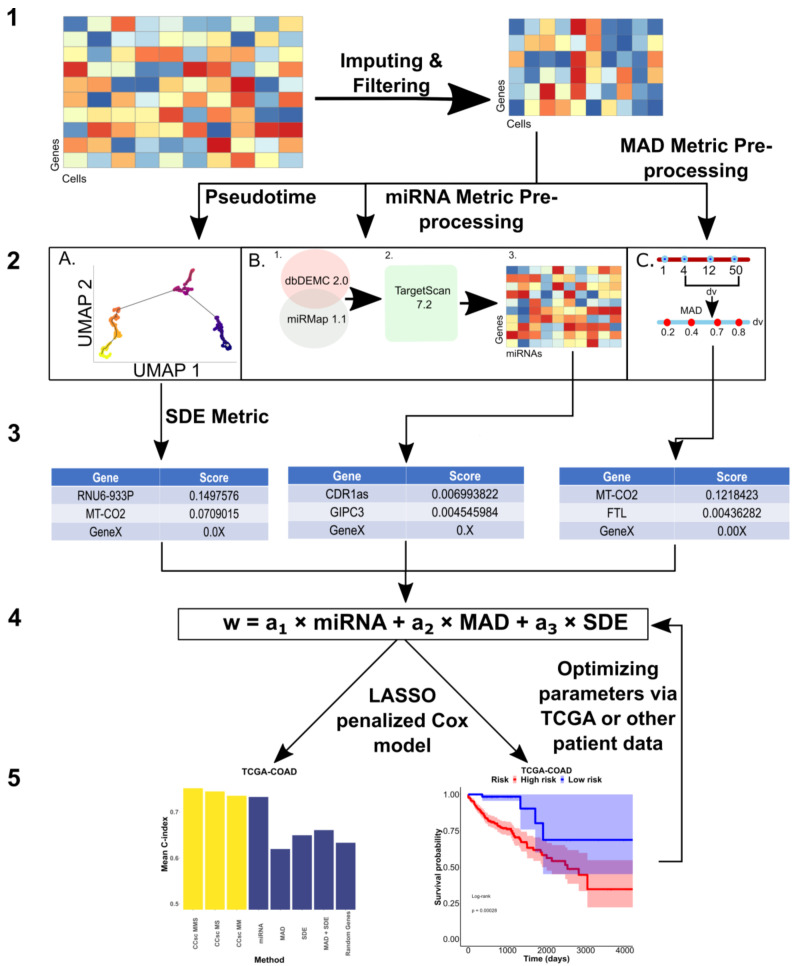
Overall Design of CCsc. CCsc is composed of 5 steps. (**1**) Obtaining pre-processed scRNA-seq matrix (genes by cells) and performing imputation and denoising via the MAGIC package and then filtering for low abundance samples. (**2**) Using filtered, denoised, and imputed scRNA-seq matrix to perform metric dependent pre-processing steps. (**A**) For the SDE metric this involves ordering the scRNA-seq matrix by pseudotime based on the expression of the *VIM* marker. (**B**) For the miRNA metric, this involves sending the matrix to our novel miRNA metric. (**C**) We calculate the MAD metric from the scRNA-seq matrix. (**3**) In this step, we generate a distinct gene list from each of our three metrics. (**4**) We integrated and merged the three separate gene lists into a single master list based on their score. From this master list, we then send *N* number of genes from the list as inputs to a LASSO penalized Cox model. The metric weights (a1, a2 and a3) are optimized via a grid search that involves the next step. (**5**) This step yields the outputs of our penalized Cox model that uses concordance index to assess our model’s accuracy. We also generate Kaplan–Meier survival curves, based on the model output, to further validate the efficacy of the genes selected by our model. Based on the Cox model outputs, we can optimize the gene weights of our linear model to achieve the best performance.

In this work, we use advances in single-cell RNA sequencing (scRNA-seq) and miRNA targeting to improve the prediction of survival outcomes for CRC patients. We develop an integrated gene prioritization method that combines miRNA–mRNA binding and target expression data (Figure 1). We show that using miRNAs and scRNA-seq provides better a predictive performance than other methods. Additionally, our method determined markers that have previously been found to be associated with other types of cancer. Finally, we show that our method can be used on other large cancer datasets to potentially find novel biomarkers and improve survival prediction accuracy.

## 2. Materials and Methods

### 2.1. Model Overview

The general pipeline that we develop in this study is called Colon Cancer Single-cell (CCsc). CCsc aims to prioritize genes for the input of a LASSO penalized Cox model. Gene prioritization integrates the rank of genes targeted by CRC-specific miRNAs and the ranks revealed by the scRNA-seq data of relevant tumors. We use one miRNA-based metric and two scRNA-seq-based metrics to construct a linear model with metric weights that are further optimized in the subsequent step, using bulk RNA-seq data from patients with survival data.

CCsc combines the outputs of three different metrics to get a final ranked list of genes. The metrics used are the interaction scores from the different miRNAs and the genes that they bind to (miRNA), the Median Absolute Deviation (MAD) of gene expression profiles, and the inference of switch-like differential expression along single-cell trajectories of genes in different stages of EMT (SDE) (R Core Team, Austria, 4.2.2) (University of Oxford, England, 1.22.0) [13,14].

### 2.2. miRNA Metric for Ranking Genes

The miRNA metric involves first obtaining the overlap of two databases (miRmap and dbDEMC); dbDEMC contains many experimentally validated microRNAs for the cancer of interest (Computational Genetics Group, China, 2.0) (Zdobnov Group, Switzerland, 1.2.0) [15,16]. We took the intersection of the cancer-specific miRNAs from dbDEMC with all of the miRNAs in miRMap. dbDEMC has collated 2224 differentially expressed miRNAs from 36 different cancer types. The miRNAs common to the dbDEMC cancer of interest and present in miRMap were then submitted to TargetScan to acquire the top-ranked genes that interact with each miRNA (Whitehead Institute for Biomedical Research, Cambridge, MA, USA, 7.2.0). The optimal number of miRNAs and target genes were determined later. We created a g × m matrix, where g is the number of genes, and m is the number of microRNAs based on the interaction data from TargetScan. Each matrix entry is either 0 for a non-interacting pair or 1 for an interacting pair. We summed the score of each gene and ranked them from highest to lowest, and returned this list of ranked genes to be used in our combined linear model (Figure S1), i.e., genes were ranked based on their total numbers of interacting microRNAs in this metric. We did this for all shared miRNAs for the cancer of interest.

### 2.3. Pre-Processing scRNA-Seq Data

Gene ranking in CC Single-cell (CCsc) has three metrics, two of which depend on processed scRNA-seq data. The single-cell dataset comes from 11 primary colorectal tumors in [17]. Briefly, the study used the 11 tumors and matched normal mucosal tissue to test an algorithm (reference component analysis) to improve clustering accuracy and elucidate new colorectal cancer subtypes [17]. This single-cell data is the foundation for both the median absolute deviation and the inference of the switch-like differential expression along single-cell trajectories (MAD and SDE) metrics. We filtered the single-cell data set to include only those genes expressed in at least 10 cells. Next, we normalized the library size with the library.size.normalize command from the phateR package (Krishnaswamy Lab, New Haven, CT, USA, 1.0.7). This command performs normalization on input data, so that the sum of the expression values for each cell sums to 1, then returns the normalized matrix to the metric space using the median UMI count per cell, effectively scaling all cells as if they were sampled evenly. We then took the square root of the dataset to transform the data, but avoid the instabilities and pseudo counts needed when taking the log of the dataset, following best practices from [18]. Next, we used the MAGIC package to denoise scRNA-seq data and imputed the missing gene expression profiles (Krishnaswamy Lab, New Haven, CT, USA, 2.0.3) [19]. Briefly, it calculates a cell–cell distance matrix in reduced dimensions. An adaptive Gaussian kernel converts the distance matrix to a cell–cell affinity (similarity) matrix. Additional steps are used to create a Markov transition matrix. The denoised scRNA-seq matrix is created by multiplying the exponentiated Markov transition matrix by the gene expression matrix.

### 2.4. Inferring EMT-Based Pseudotime

The SDE metric requires an ordering of cells that represents an activation or deactivation process. Despite the absence of time-series information, we inferred the pseudotime ordering of cells in the scRNA-seq data [20]. We used a scRNA-seq dataset for colorectal tumors that primarily contain epithelial cells [17]. Epithelial-mesenchymal transition is a known driver for epithelial plasticity and tumor progression for colorectal cancer and several other cancer types [17,21,22,23,24]. There, we used the expression levels of a specific mesenchymal signature gene, *VIM*, as an approximation for cells progressing through EMT [24,25,26]. In the scRNA-seq dataset, we found that the expression levels of most epithelial (E) genes were negatively correlated with pseudotime, whereas those of mesenchymal (M) genes were positively correlated with pseudotime (Figure S2) [23,24]. This suggests a reasonable pseudo-temporal ordering of epithelial tumor cells. It should be noted that, here, we do not use pseudo-temporal ordering to infer trajectories of cell state transitions during development. We, therefore, do not analyze the connectivity of cell state attractors (cell types). Instead, the ordering allows us to analyze the variations in expression for all genes in a relatively uniform framework of cell states (see the description of the SDE method below).

### 2.5. MAD Metric for Ranking Genes

MAD is calculated with

(1) $$m_{g}=median\left(e_{1},e_{2},\;\ldots \;,e_{c}\right)$$

(2) $$MAD\left(g\right)=median\left(\;\left|e_{1}-m_{g}\right|,\;\left|e_{2}-m_{g}\right|,\;\ldots ,\left|e_{c}-m_{g}\right|\right)$$

where $e_{i}$ represents the expression level of a gene g in cell i, and c is the number of cells.

### 2.6. Inference of Switch-Like Differential Expression along Single-Cell Trajectories (SDE) Metric for Ranking Genes

To calculate SDE, we used the R package switchde, which estimates the differentiation of switch-like genes in different stages of EMT. It defines a sigmoid function

(3) $$f\left(t_{c};\mu_{g}^{0};\;k_{g};\;t_{g}^{0}\right)=\frac{2\mu_{g}^{0}}{1+\exp\left(-\kappa_{g}\left(t_{c}-t_{g}^{0}\right)\right)}$$

to fit the profile of a gene g concerning a pseudotime. In Equation (2), $\mu_{g}^{0}$ corresponds to the average peak expression; $\kappa_{g}$ is the activation nonlinearity; $t_{c}$ is the active pseudotime of g in cell c; and $t_{g}^{0}$ is the offset of the activation. Intuitively, $\kappa_{g}$ represents how quickly the gene g is up or downregulated along the pseudotime and is used as the metric score. Note that $\kappa_{g}$ may reflect how genes are switched on or off dynamically due to transcriptional bursting [27], but it can also include other sources of variability of gene expression, such as mutations and post-transcriptional regulations [28,29]. Therefore, the parameter should be viewed as a characteristic variational pattern of gene expression across a cell population in the pseudo-temporal trajectory, common to all genes.

### 2.7. Overall Gene Prioritization Scoring

To make ranges of gene scores consistent across the three metrics (MAD, SDE and miRNA), each gene’s final score for each metric was normalized to be between 0 and 1, by dividing its raw score by the sum of the score of all genes for the metric. Next, we combined the scores from the three metrics using the function

(4) $$w=a_{1}w_{\mathrm{SDE}}+a_{2}w_{\mathrm{MAD}}+a_{3}w_{\mathrm{miRNA}}$$

where $w_{\mathrm{SDE}}$, $w_{\mathrm{MAD}}$ and $w_{\mathrm{miRNA}}$ are the normalized metric scores. Parameters $a_{1}$, $a_{2}$ and $a_{3}$ are metric weights for combining the metric scores into the overall score w. If a gene does not have a score for miRNA metric due to the lack of miRNA targeting information, its score is assumed to be zero. We ranked the genes based on overall scores, and we selected the genes with high scores (see details later) for the subsequent analysis. The values of $a_{1}$, $a_{2}$ and $a_{3}$ were determined by a grid search with an interval of 0.1, and the constraint of the sum of these parameters is 1. The performance of the grid search is based on the Cox model and the concordance index described below.

### 2.8. LASSO Regularized Cox Model

To build prognostic models and to further select important genes from the prioritized lists, we used bulk RNA-seq data and the associated patient survival data from the Cancer Genome Atlas (TCGA) or cBioPortal for Cancer Genomics. We used a LASSO regularized Cox model to determine the concordance index and top-performing coefficients. This regularization uses the L1 lasso penalty. This allows the number of coefficients to be constrained based on the value of the penalty weight parameter λ that we use. The regularization was used to avoid the overfitting of our models. Regularizations help to address overfitting and make Cox models more interpretable. The LASSO regularization, which involves finding a subset of predictors which give a model’s best overall performance, is used here to improve the interpretability of our model [30,31,32,33]. We used the glmnet package in R to perform all the LASSO regularized Cox-model fittings (Hastie Lab, Stanford, CA, USA, 4.1-6) [34,35].

### 2.9. Concordance Index (C-Index)

The concordance index (C-index) is the primary metric for assessing our method’s effectiveness. This metric is analogous to the area under the curve–receiver–operator characteristic, but is applied specifically for survival analysis situations. It is calculated by

(5) $$C-index=\frac{\sum \sum_{i<j}\left[I\left(t_{i}<t_{j}\right)I\left(r_{i}>r_{j}\right)I\left(\delta_{i}\;\equiv 1\right)+I\left(t_{i}>t_{j}\right)I\left(r_{i}<r_{j}\right)I\left(\delta_{j}\;\equiv 1\right)\right]}{\sum \sum_{i<j}\left[I\left(t_{i}<t_{j}\right)I\left(\delta_{i}\;\equiv 1\right)+I\left(t_{i}>t_{j}\right)I\left(\delta_{j}\;\equiv 1\right)\right]}.\;$$

The C-index is equal to the concordance probability *p* (*r_i_* > *r_j_*|*z_i_* < *z_j_*) for a randomly selected pair of patients *I* and *j*. Unfortunately, we cannot observe the potential survival time for some patients who are lost to follow-up or are event free at the end of a study (right censored). Given this, the observed survival time *t_i_* = *min* (*z_i_*, *c_i_*), where *c_i_* is the potential right censoring time; *δ_i_* is the censoring status. An event (e.g., death) is when *δ_i_* = 1. *I ()* is the indicator function, and *r* is equal to the risk score for patients *i* and *j,* respectively.

Essentially, this metric assesses the ability of a set of input predictors to accurately judge whether a patient with a particular risk score will get cancer in a specific period. Specifically, the C-index judges whether a model has discriminatory power and accurately ranks the patient’s survival time when considering their calculated risk scores. In an ideal case, a model would ideally separate all patients based on their risk score into their correct group and would have a performance of 1. A model with a C-index of 0.5 is classified as a random predictor [36].

### 2.10. Kaplan-Meier Survival Analysis

The other primary output of our model is a Kaplan–Meier risk estimator (Terry Therneau, Rochester, MN, 3.4-0) [37]. This metric attempts to determine how well a set of inputs in a model can correctly stratify patients in our datasets as high risk or low risk, based on their gene expression profiles. The survival probability s at time t is given by

(6) $$s_{t}=\frac{\mathrm{Number}\;\mathrm{of}\;\mathrm{subjects}\;\mathrm{living}\;\mathrm{at}\;\mathrm{the}\;\mathrm{start}\;-\;\mathrm{Number}\;\mathrm{of}\;\mathrm{subjects}\;\mathrm{who}\;\mathrm{died}}{\mathrm{Number}\;\mathrm{of}\;\mathrm{subjects}\;\mathrm{living}\;\mathrm{at}\;\mathrm{the}\;\mathrm{start}}$$

The Kaplan–Meier survival analysis uses the log-rank test to assess whether the high and low-risk groups’ survival times differ statistically. The statistic is given by

(7) $$\mathrm{Log}-\mathrm{rank}\;\mathrm{test}\;\mathrm{statistic}=\frac{{\left(O-E\right)}^{2}\;}{E}$$

O is the total of the observed events and E is the total of the expected events. For each event of interest (death), we calculate the number of deaths observed and the number of deaths expected, if there was really no difference between our groups. This calculation is performed for both the high and low-risk groups in our data. We then sum the number of observed and expected events to get O and E in (5). If a survival time is censored, that particular individual is considered to be at risk of dying in the week of the censoring, but not in subsequent weeks [38].

A *p*-value < 0.05 is statistically significant for our Kaplan–Meier survival analysis.

### 2.11. Datasets

The datasets in this study include several single-cell and bulk RNA-seq datasets, and we used several scRNA-seq based and bulk RNA-seq based methods either for constructing our model or benchmarking. The scRNA-seq data was obtained from a previous study on combined tumor samples of 11 colorectal cancer patients [17]. The bulk RNA-seq data of colon and rectal cancer were obtained from the Cancer Genome Atlas (TCGA), which were used to train the Cox models. The colon cancer dataset includes 461 cases, and the rectal cancer dataset includes 172 cases. The TCGA datasets can be accessed through the Genome Data Commons web portal or the TCGAbiolinks R package (https://portal.gdc.cancer.gov, accessed on 1 December 2022) [39]. The additional bulk dataset from the cBioPortal for Cancer Genomics contains 79 cases and can be accessed through the cBioPortal web interface (https://www.cbioportal.org, accessed on 1 December 2022) [40]. The scRNA-seq data can be found at GEO under accession GSE81861.

### 2.12. Implementation

We have implemented our metric and model in R on GitHub (https://github.com/compbiolover/CC-Singlecell) (Accessed 1 December 2022). All code and datasets used in this manuscript are available there.

## 3. Results

**Figure 2:**
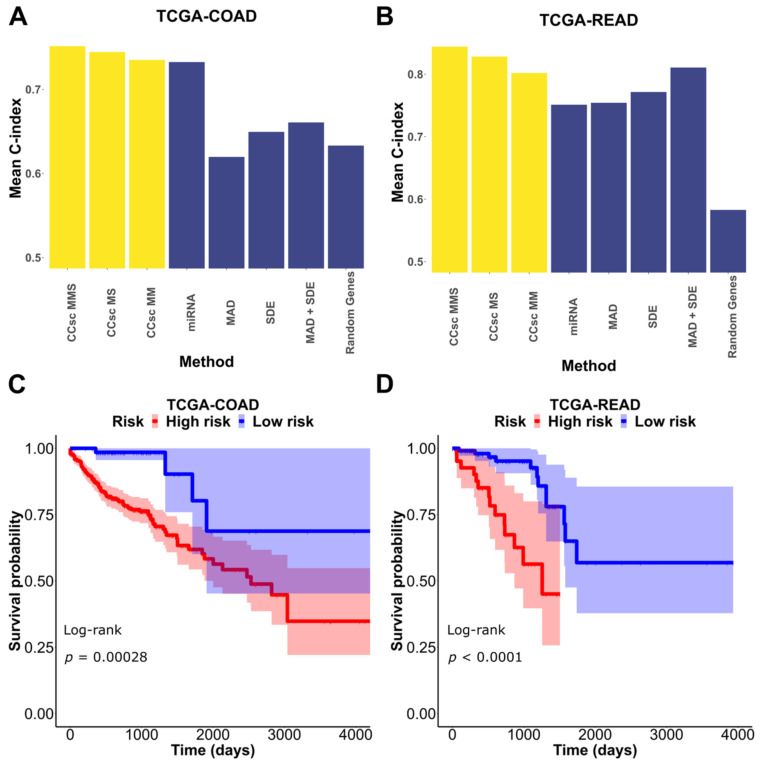
Combination of metrics is better than any individual metric. (**A**) We tested each of our models’ metrics and compared them to CCsc MS, CCsc MM, and CCsc MMS and a set of randomly selected genes, equal to the number of genes used by our method on the TCGA-COAD dataset. (**B**) We made the same comparison on the TCGA-READ dataset. We show that integrating the lists of ranked genes from each metric provides better performance than any one individually. (**C**) Kaplan–Meier estimate based on our model’s top set of predictors for the TCGA-COAD dataset. (**D**) Kaplan–Meier estimate based on our model’s top set of predictors for the TCGA-READ dataset. The shaded regions represent 95% confidence intervals of the survival estimates. The *p*-value threshold for significance is < 0.05.

CCsc has three metrics to prioritize genes for prognostic predictions: miRNA (based on disease-related miRNAs), MAD (based on variability of gene expression), and SDE (based on switch-like behaviors of gene expression) (see Materials and Methods for details). To evaluate these three metrics, we tested multiple versions of CCsc. They include CCsc miRNA + MAD (CCsc MM), CCsc miRNA + SDE (CCsc MS), and CCsc miRNA + MAD + SDE (CCsc MMS) (Figure 2A,B). To prioritize genes, we used a recent scRNA-seq dataset for colorectal cancer cells [17], and we used TCGA-COAD (Colon Adenocarcinoma) and -READ (Rectum adenocarcinoma) datasets for prognostic performance evaluations (see Methods for details). We compared each combination’s mean 10-fold cross-validated C-index, while holding the number of genes constant. We did this for both TCGA-COAD and TCGA-READ. Based on our test results (Figures S3 and S4), we concluded that combining all three metrics gave us the best mean concordance index performance across both datasets. In addition, we found that CCsc MMS could separate high-risk from low-risk patients for both the TCGA-COAD and TCGA-READ datasets (Figure 2C,D). Taken together, the combinations of all three metrics (i.e., CCsc MMS) perform better than other choices, suggesting the importance of prioritizing genes based on both miRNA-targeting information and the summary statistics of expression. We therefore used CCsc MMS for our subsequent analyses.

**Figure 3:**
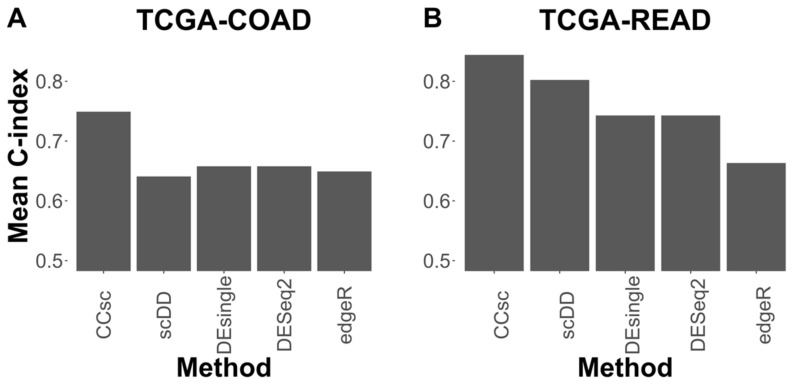
CCsc MMS Outperforms Other Methods. (**A**) We tested CCsc against several well-established tools on the TCGA-COAD dataset. We compared the mean concordance index performance of CCsc with its ideal weighting to DESeq2, edgeR, scDD, and DEsingle. We gave each method its optimal number of genes and ran each with its recommended settings, according to their respective best practices. (**B**) Same comparison but on the TCGA-READ dataset. We show that CCsc outperforms single-cell methods (scDD and DEsingle) and that bulk RNA-seq methods can be optimized for single-cell RNA-seq data (DESeq2, edgeR).

To further evaluate the importance of miRNA-targeting in gene prioritization, we compared the mean 10-fold cross-validation performance of CCsc MMS to that of several other methods that select genes based on differential expression. For this comparison, we chose two methods initially designed for bulk RNA-seq analysis, but that have been updated to use single-cell data (DESeq2 and edgeR), and two methods designed specifically for scRNA-seq data (scDD and DEsingle). For both TCGA-COAD and TCGA-READ, CCsc MMS has the best mean 10-fold performance compared to all other methods. In the case of COAD, CCsc has a markedly better performance (0.7514 vs. ~0.65 for all other methods) and a slightly higher performance with READ (0.8442 vs. 0.8023 for scDD) (Figure 3). 

**Figure 4:**
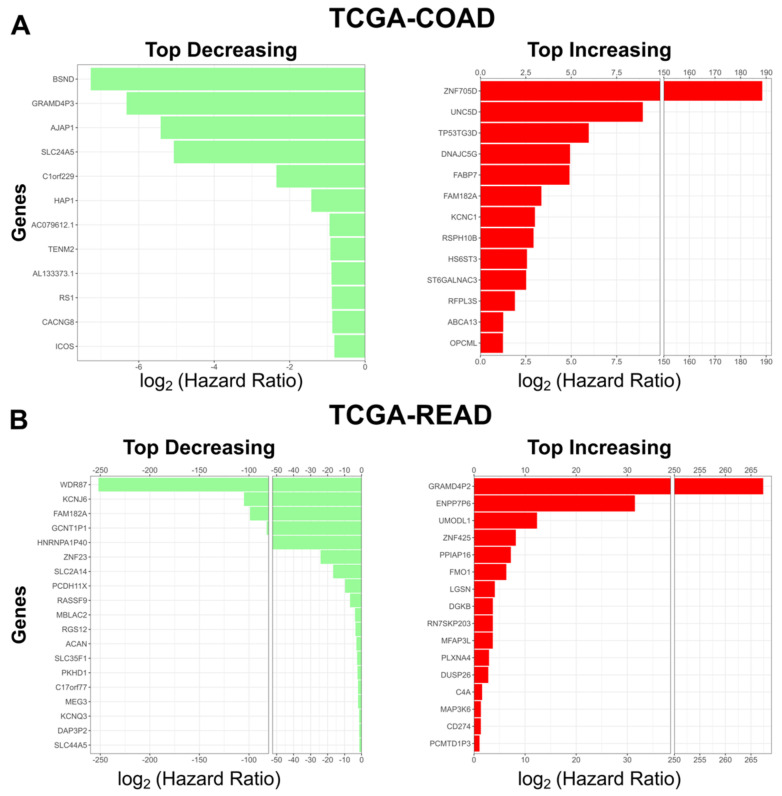
Top coefficients identified by our model. (**A**) Top risk decreasing (green, left) and top risk increasing (red, right) genes identified by our Cox model in TCGA-COAD. (**B**) Top risk decreasing genes (green, left) and top risk increasing (red, right) genes identified by our Cox model in TCGA-READ. For risk increasing genes the hazard ratio threshold is > 2. For risk decreasing genes the hazard ratio threshold is < 0.5.

We then asked what the top predictors in the model were. We found 13 genes that had a hazard ratio > 2 for COAD, and 16 such genes for READ. Next, we found 12 genes that had a hazard ratio < 0.5 for COAD, and 19 genes for READ (Figure 4A,B). Many of the genes that increase patient risk (12/13 COAD and 11/16 READ) were previously implicated in cancer progression (Table 1 and Table 2).

Genes such as *OPCML* have been found to be silenced in tumors, and when reactivated, they lead to cancer tumor inhibition. Several of the genes found to increase patient risk in CRC by our model have been found to be silenced in other types of cancers. Additionally, we examined the genes with the smallest hazard ratios to see which of our model’s genes might be indictive of better clinical outcomes. We found several genes that were associated with a substantial reduction in patient risk and have been associated with a decreased patient risk in various cancers (Table 3 and Table 4). Of the top predictors in READ, without the support of the literature, were all annotated as pseudogenes. However, given the large hazard ratio of these genes in our model, our results suggest that it either plays a role in the pathological process or serves as a signal for cellular changes that lead to cancer progression.

Next, we sought to see what pathways and cellular processes were influenced by these genes. We submitted the genes from Figure 4 (10 for COAD and 13 for READ) separately to Reactome version 3.7 (https://reactome.org) (Accessed 1 December 2022), and identified pathways related to *NOTCH3* signaling and Flavin-containing monooxygenases (FMO) oxidizing nucleophiles (Table 5 and Table 6). The pathways impacted by the top genes have been implicated in CRC progression and metastasis [92,93,94,95,96,97].

## 4. Discussion

CRC has proven to be a complex disease that, despite the marked research focus and improvements in biomarker detection, still has many open questions. Based on the important roles that miRNAs play in the regulation of many cellular processes, including processes related to CRC, we developed a novel metric that uses the prevalence of miRNA-target interactions to prioritize genes for prognostic models. In conjunction with the switchde and MAD methods, we create an integrated model, CCsc MMS, to improve the ability to accurately predict colorectal patient survival. We show this performance improvement across multiple large datasets related to colon and rectal cancer. We show that our model outperforms multiple existing methods, including DESeq2, DEsingle, scDD, and edgeR, which have been developed for identifying differentially expressed genes with both bulk and single-cell RNA seq data. The improvement is facilitated by the incorporation of the LASSO regularization. This regularization allows the simplification of the model and avoids overfitting. When we examined the weight of these genes, we found that many of our model’s top performers are implicated in various types of cancers, including CRC. We then performed pathway analysis with Reactome and found that this handful of genes is enriched in pathways related to NOTCH3 signaling and potassium channels, which are important in CRC. We attempted pathway enrichment analysis for both the top genes associated with higher patient risk and those that were associated with lower patient risk. The top markers in the lower risk analysis for both TCGA-COAD and TCGA-READ did not meet our significance criteria, and hence no pathways were identified for the lower patient risk genes. In addition, we found that our method uses comparable numbers of active genes to those from these existing methods, while giving better performance (Figure S5). We also found that our approach had a satisfactory performance for non-TCGA CRC datasets (Figure S6). Finally, we asked if any of the top predictors were known to be regulated by miRNAs. We found that many of the genes most impactful on patient survival are directly or indirectly regulated by miRNAs in disease settings (Table S1). In addition, we quantified the mean expression of all the genes in each of our COAD and READ signatures and compared them to the overall mean across genes for each of the datasets. For both COAD and READ, we observed that all genes in our signature sets were well below the mean expression of all genes in the datasets (Figure S7).

## 5. Conclusions

In conclusion, we developed a novel metric based on miRNA-gene target interactions that improved an integrated model’s predictive performance in CRC. We demonstrated that our method, CCsc MMS, outperforms existing methods and that we have a more interpretable model by using a LASSO regularization. We show that CCsc MMS is a valuable method for predicting the survival of CRC patients and offering an interpretable and insightful way to examine the most important genes in a large data context. We show that many of these largest coefficients are enriched for various aspects of NOTCH3 signaling, potassium, and overall metabolism, which has been shown to play an important role in CRC.

## Figures and Tables

**Table 1:** Top Risk Increasing Genes Identified by TCGA-COAD Cox model: List of the top risk increasing genes identified by our Cox model on the TCGA-COAD dataset.

Gene	Literature Support
*ZNF705D*	[41]
*UNC5D*	[42]
*TP53TG3D*	[43,44]
*ST6GALNAC3*	[45,46]
*RSPH10B*	[47]
*KCNC1*	[48,49]
*HS6ST3*	[50,51]
*FAM182A*	None
*FABP7*	[52,53]
*DNAJC5G*	[54]
*ABCA13*	[55]
*OPCML*	[56]
*RFPL3S*	[57]

**Table 2:** Top Risk Increasing Genes Identified by TCGA-READ Cox model: List of the top risk increasing genes identified by our Cox model on the TCGA-READ dataset.

Gene	Literature Support
*ZNF425*	[58]
*UMODL1*	[59]
*RN7SKP203*	Pseudogene
*PPIAP16*	Pseudogene
*PLXNA4*	[60,61]
*MFAP3L*	[62,63]
*LGSN*	[64]
*GRAMD4P2*	Pseudogene
*FMO1*	[65]
*ENPP7P6*	Pseudogene
*DUSP26*	[66,67]
*DGKB*	[68,69]
*MAP3K6*	[70]
*CD274*	[71]
*C4A*	[72]
*PCMTD1P3*	Pseudogene

**Table 3:** Top Risk Decreasing Genes Identified by TCGA-COAD Cox model: List of the top risk decreasing genes identified by our Cox model on the TCGA-COAD dataset.

Gene	Literature Support
*AJAP1*	[73]
*SLC24A5*	None (potassium-dependent sodium/calcium exchanger)
*CACNG8*	[74]
*C1orf229*	None
*GRAMD4P3*	Pseudogene
*ICOS*	[75]
*HAP1*	[76]
*TENM2*	[77]
*AC079612.1*	[78]
*AL133373.1*	None
*BSND*	[79]
*RS1*	[80]

**Table 4:** Top Risk Decreasing Genes Identified by TCGA-READ Cox model: List of the top risk decreasing genes identified by our Cox model on the TCGA-READ dataset.

Gene	Literature Support
*FAM182A*	None
*SLC35F1*	[81]
*RGS12*	[82]
*PKHD1*	[83]
*GCNT1P1*	Pseudogene
*KCNJ6*	None (potassium channel)
*RASSF9*	[84]
*DAP3P2*	Pseudogene
*WDR87*	None
*KCNQ3*	[85]
*PCDH11X*	[86]
*MEG3*	[87]
*MBLAC2*	[88]
*SLC44A5*	None (Predicted to enable transmembrane transporter activity)
*HNRNPA1P40*	Pseudogene
*ZNF23*	[89]
*ACAN*	[90]
*SLC2A14*	[91]

**Table 5:** Statistically Significantly Enriched COAD Pathways: Most enriched pathways influenced by the genes with the largest hazard ratios associated with increased risk in our TCGA-COAD Cox model. *p*-value threshold <0.05 FDR. Hazard ratio threshold >2.

Pathway	Literature Support	FDR *p*-Value
NOTCH3 Intracellular Domain Regulates Transcription	[92]	1.98 × 10^−2^
Voltage gated Potassium channels	[93]	1.98 × 10^−2^
Signaling by NOTCH3	[94]	2.62 × 10^−2^

**Table 6:** Statistically Significantly Enriched READ Pathways: Most enriched pathways influenced by the genes with the largest hazard ratios are associated with increased risk in our TCGA-READ Cox model. *p*-value threshold <0.05 FDR. Hazard ratio threshold >2.

Pathway	Literature Support	FDR *p*-Value
Activation of C3 and C5	[98]	1.48 × 10^−3^
STAT3 nuclear events downstream of ALK signaling	[99]	4.74 × 10^−3^
Signaling by ALK	[100]	1.72 × 10^−2^
FOXO-mediated transcription of oxidative stress, metabolic and neuronal genes	[96]	1.72 × 10^−2^

## Data Availability

All computer code of this study can be found at https://github.com/compbiolover/CC-Singlecell.

## Supplementary Materials

The following supporting information can be downloaded at: https://www.mdpi.com/article/10.3390/cells12020228/s1, Figure S1: miRNA metric; Figure S2: Pseudo-temporal ordering analysis; Figure S3: LASSO regularization gives the best performance; Figure S4: Determination of Ideal miRNA and miRNA target values; Figure S5: CCsc MMS Uses Comparable Gene Set Size to Existing Methods; Figure S6: CCsc MMS Outperforms other Methods in Non-TCGA CRC Data; Figure S7: Highly Ranked Gene Targets Show Reduced Expression Level. Table S1: Genes Identified by Model Regulated by miRNA. Genes are either directly or indirectly regulated by miRNAs.
