# Supplementary material for: Using Single-Cell RNA Sequencing and MicroRNA Targeting Data to Improve Colorectal Cancer Survival Prediction
Source: Cells. 2023 Jan 5;12(2):228. doi: 10.3390/cells12020228 (PMC9856396; doi:10.3390/cells12020228)
Supplement: Supplementary file 1 [file cells-12-00228-s001.zip › cells-2104616-supplementary.pdf]

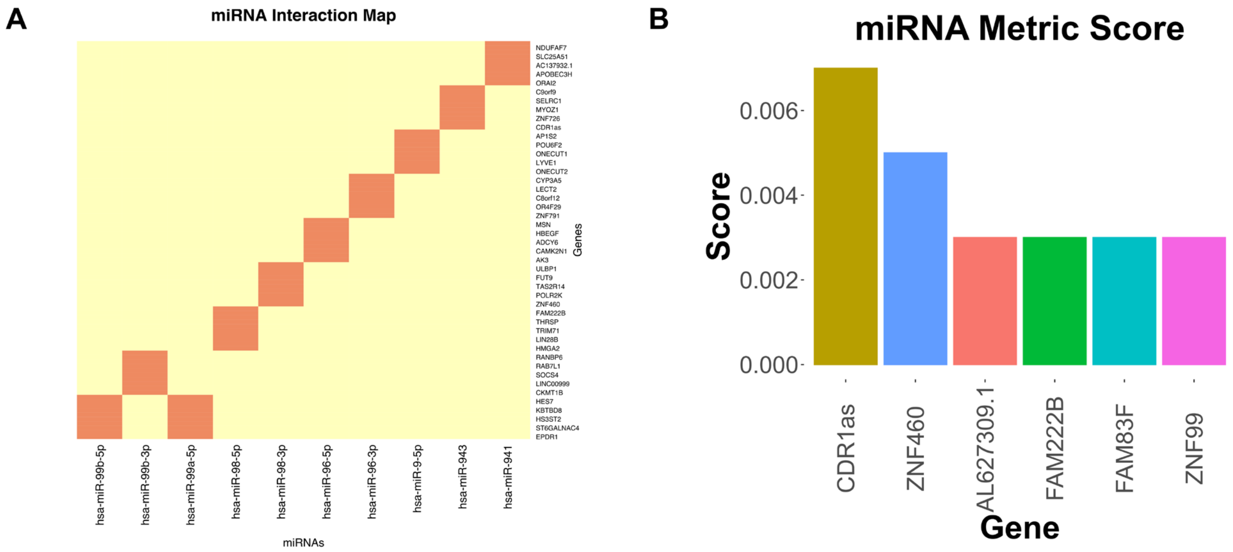


**Figure S1**. miRNA metric (**A**) Heatmap depicting the interactions between the experimentally validated miRNAs and their targets in READ data. Here we show a small subset of the genes and miRNAs that make up our miRNA metric for the TCGA-COAD dataset. These were acquired by first taking the overlap of the miRNAs from miRMap and dbDEMC 2.0. Then these common miRNAs were submitted to the TargetScan database, and these miRNAs' top *N* gene targets were returned. The number of times each miRNA interacted with each gene was then calculated by simply increasing the number for a gene by one if it interacted with a particular miRNA. Red is high binding; blue is low binding. Once all miRNA-target pairs were evaluated, the genes were ranked from the greatest number of interactions to the fewest. This score was then normalized to between 0 and 1. (**B**) Example of some of the top gene targets for the COAD dataset.

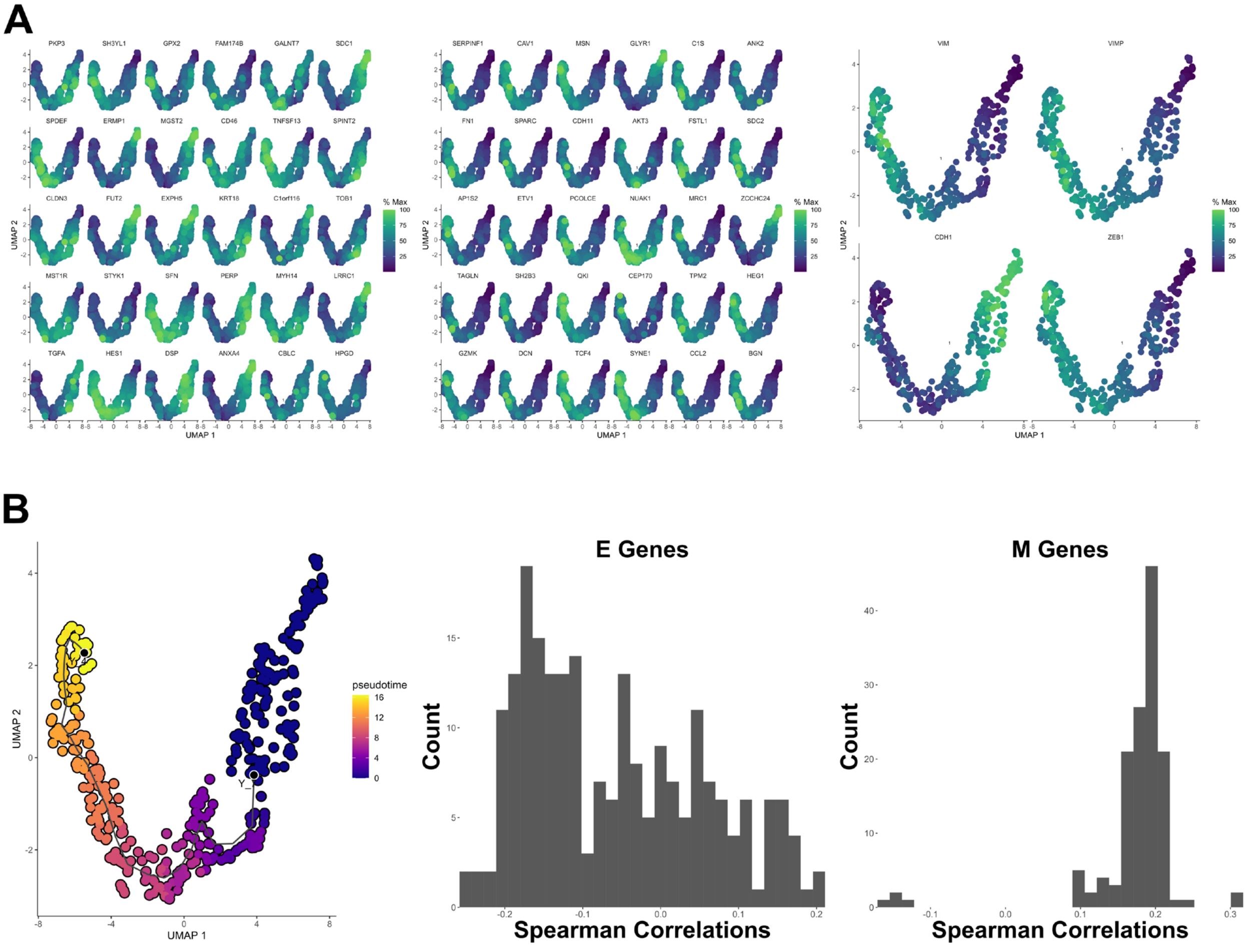


**Figure S2**. Pseudo-temporal ordering analysis. (**A)** UMAP of gene expression of 30 randomly selected epithelial (E) annotated genes (left panel). UMAP of gene expression of 30 randomly selected mesenchymal (M) annotated genes (center panel). UMAP of gene expression of 4 hallmark EMT genes that represent E (CDH1) or M (VIM, VIMP, ZEB1) gene expression states in the Li et al data (right panel). (**B)** UMAP of Li et al data colored by pseudotime values based on the gene expression profile of VIM (left). Histograms of the distributions of the Spearman correlation coefficients between gene expression levels and pseudotime values for all E annotated (center) or M annotated (right) genes.

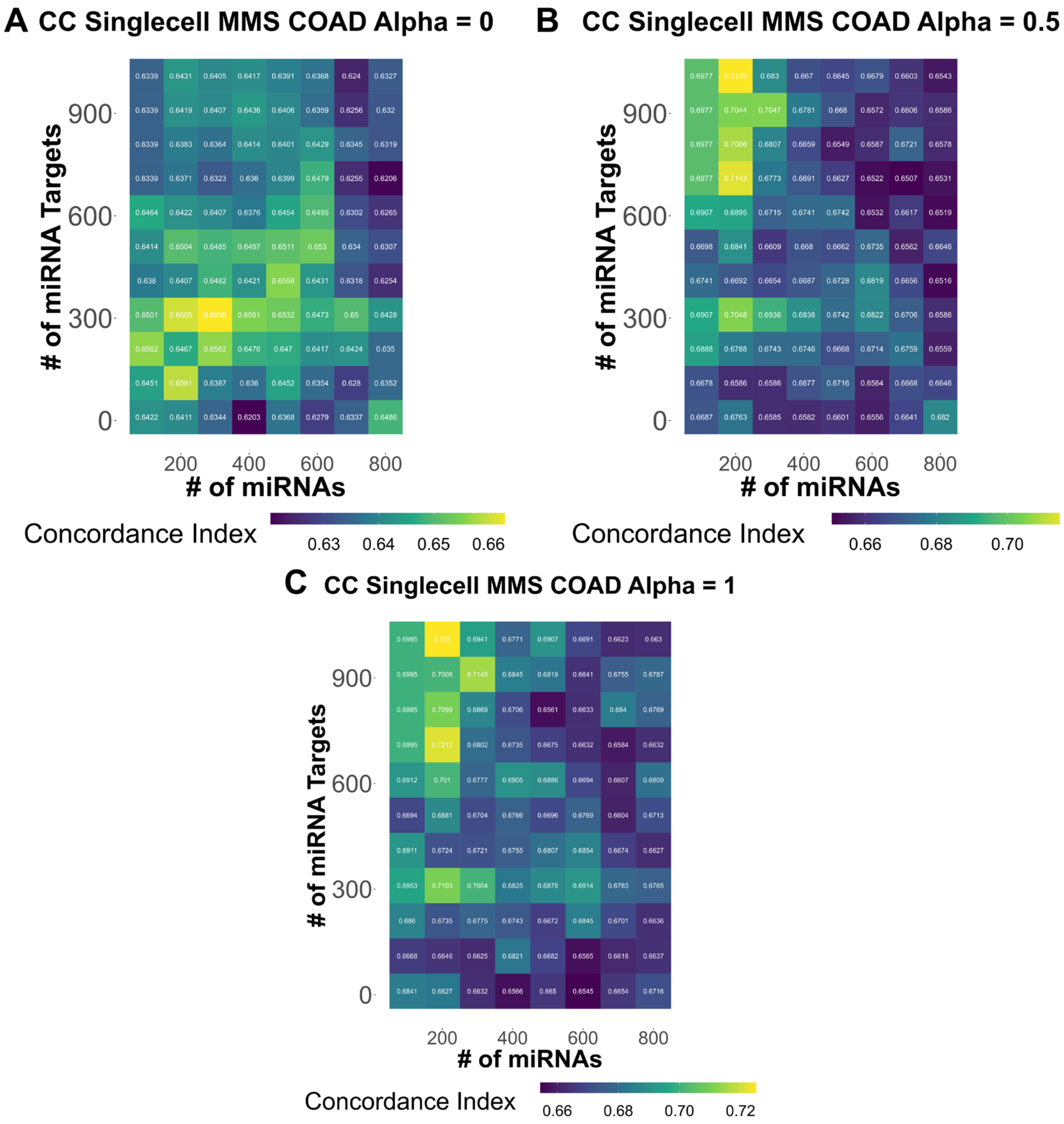


**Figure S3.** LASSO regularization gives the best performance. Heatmaps showing our grid searches at several different values of alpha for (**A**) 0, (**B**) 0.5, and (**C**) 1. We plot the mean 10-fold cv at each combination of miRNA and miRNA target number.

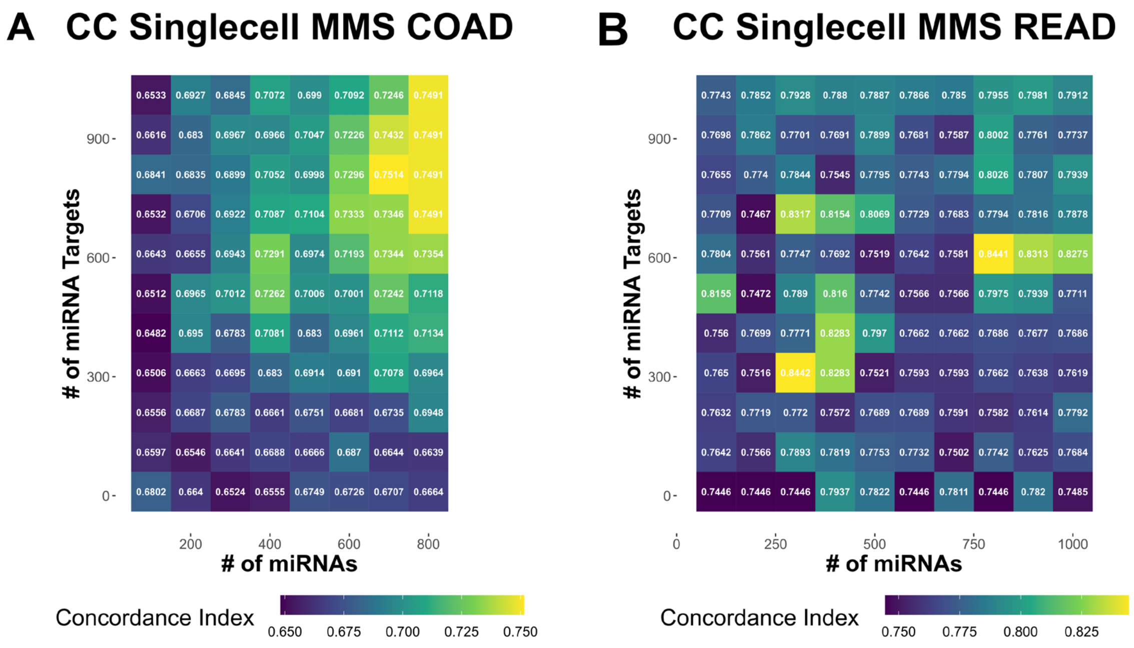


**Figure S4.** Determination of Ideal miRNA and miRNA target values. Heatmaps showing our grid searches of many combinations of miRNAs and miRNA targets for (**A)** TCGA-COAD and (**B)** TCGA-READ. We send each combination of miRNAs and miRNA targets through our pipeline, and then we send the ideal number of genes in each integrated list of genes from our model into the LASSO Cox model for TCGA-COAD and TCGA-READ datasets. The gene size for TCGA-COAD is 2,500, and the gene size for TCGA-READ is 600. We then plot the concordance index of each combination.

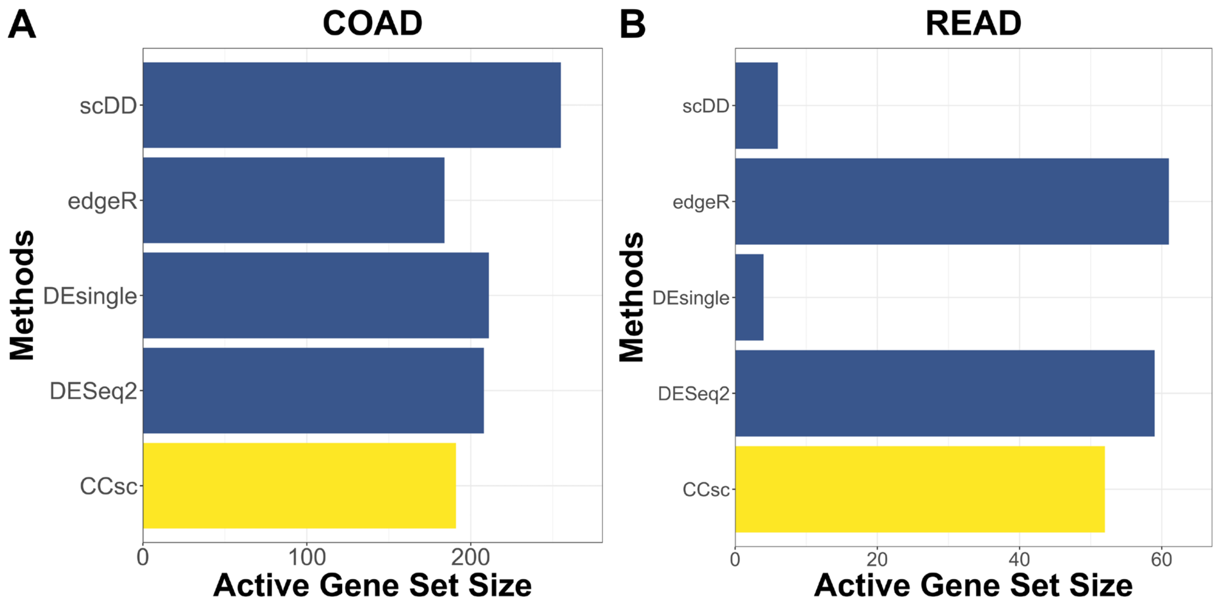


**Figure S5.** CCsc MMS Uses Comparable Gene Set Size to Existing Methods. **(A)** We ran each method at optimal gene set size and then extracted the non-zero betas from the LASSO penalized Cox model (i.e., Active genes). We see that, on average, CCsc has a much higher concordance index than other methods while using approximately the same number of genes. We also see this in TCGA-READ (**B)**

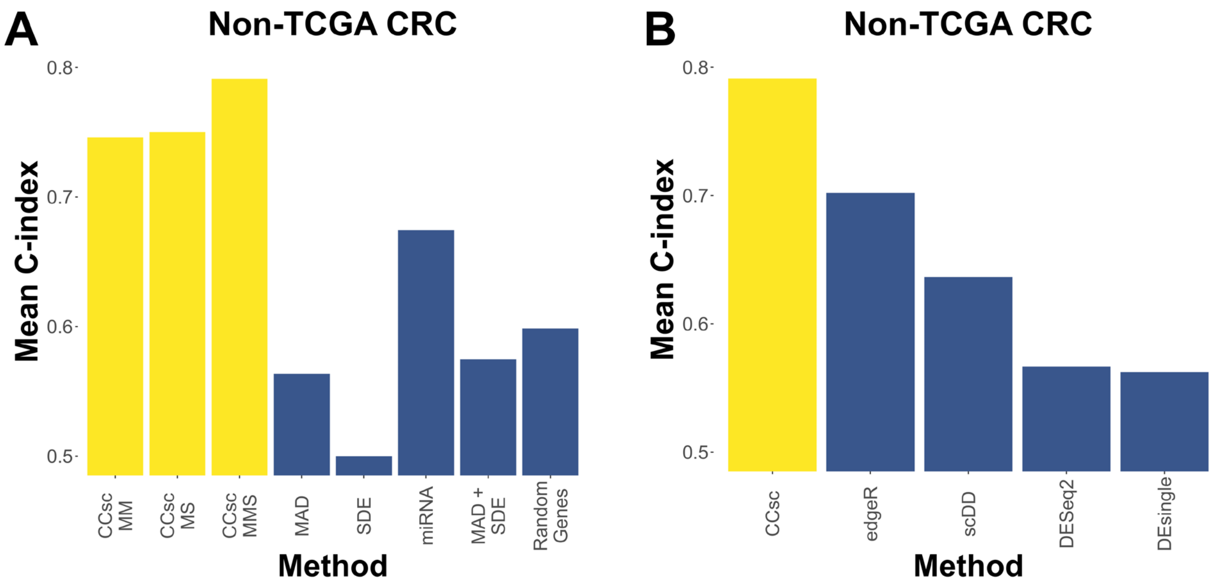


**Figure S6.** CCsc MMS Outperforms other Methods in Non-TCGA CRC Data. We performed the same analysis on a non-TCGA CRC dataset. We found that CCsc MMS continues to outperform its individual components (**A**). Additionally, it outperformed all other methods on this new dataset (**B**).

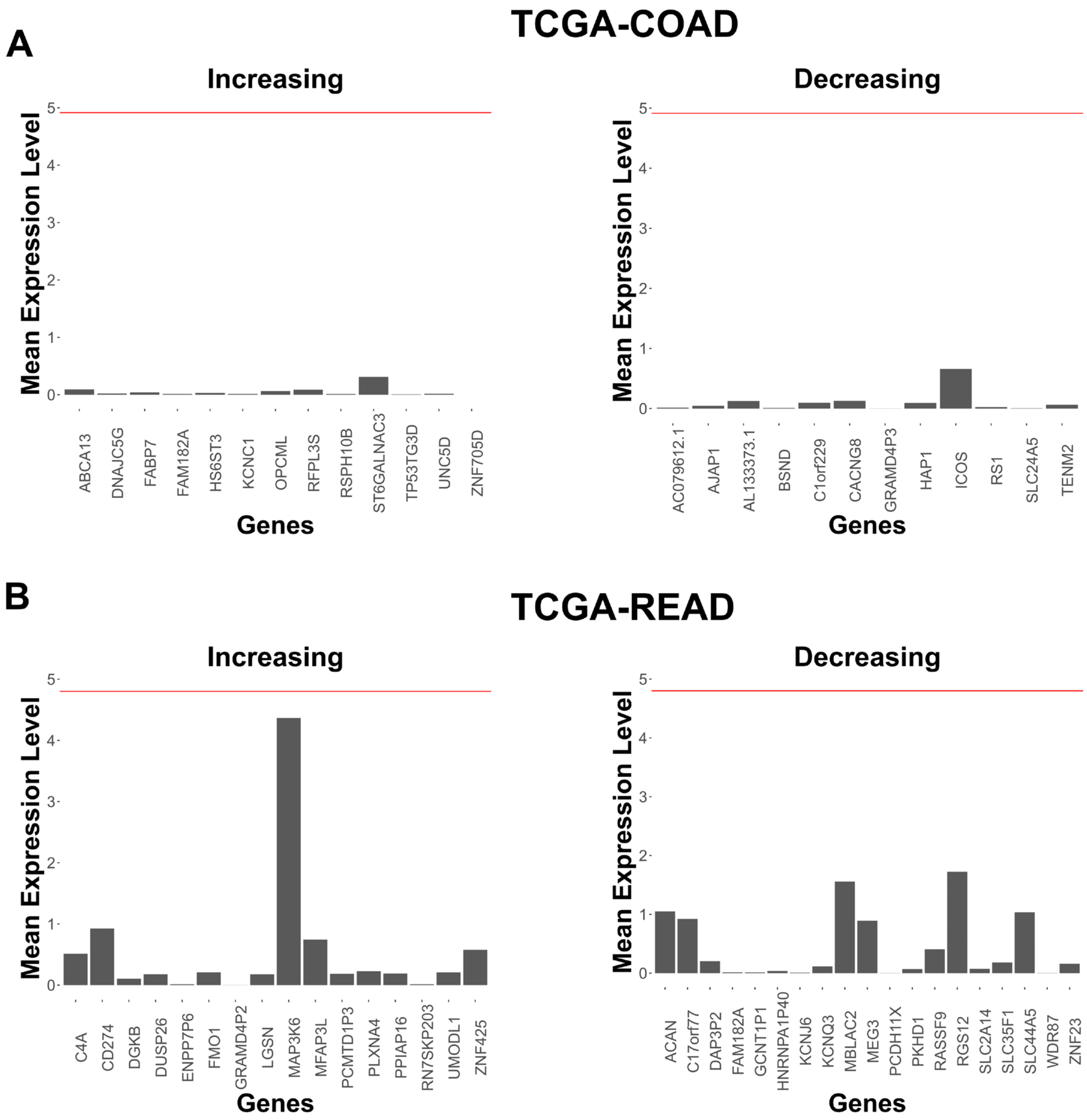


**Figure S7.** Highly Ranked Gene Targets Show Reduced Expression Level. Each of the top performing genes in our signatures showed much reduced expression compared to the mean of all genes present in the (**A**) TCGA-COAD and (**B**) TCGA-READ datasets respectively. This was true for both the top genes that were found to increase patient risk (left panels) and to decrease patient risk (right panels). Horizontal red line denotes the mean expression of each dataset.

| Gene | Directly Regulated | Indirectly Regulated | Citation |
|---|---|---|---|
| ZNF705D | N | N | N/A |
| UNC5D | Y | N | [1] |
| TP53TG3D | Y | Y | [2] |
| ST6GALNAC3 | Y | N | [3] |
| RSPH10B | N | N | N/A |
| KCNC1 | Y | N | [4] |
| HS6ST3 | Y | N | [5] |
| FAM182A | N | N | N/A |
| FABP7 | N | Y | [6] |
| DNAJC5G | N | N | N/A |
| ZNF425 | N | N | N/A |
| UMODL1 | N | N | N/A |
| RN7SKP203 | N | N | N/A |
| PPIAP16 | N | N | N/A |
| PLXNA4 | Y | N | [7] |
| MFAP3L | Y | N | [8] |
| LGSN | N | N | N/A |
| GRAMD4P2 | N | N | N/A |
| FMO1 | N | N | N/A |
| ENPP7P6 | N | N | N/A |
| DUSP26 | N | Y | [9] |
| DGKB | Y | **N** | [10] |

**Table S1.** Genes Identified by Model Regulated by miRNA. Genes are either directly or indirectly regulated by miRNAs.

## References


1. Zhu, Y.; Li, Y.; Nakagawara, A. UNC5 dependence receptor family in human cancer: a controllable double-edged sword. *Cancer Letters* **2021**, *516*, 28-35.
2. Lu, Q.; Guo, Q.; Xin, M.; Lim, C.; Gamero, A.M.; Gerhard, G.S.; Yang, L. LncRNA TP53TG1 Promotes the Growth and Migration of Hepatocellular Carcinoma Cells via Activation of ERK Signaling. *Non-coding RNA* **2021**, *7*, 52.
3. Kasper, B.T.; Koppolu, S.; Mahal, L.K. Insights into miRNA regulation of the human glycome. *Biochemical and biophysical research communications* **2014**, *445*, 774-779.
4. Gu, X.-Y.; Jin, B.; Qi, Z.-D.; Yin, X.-F. MicroRNA is a potential target for therapies to improve the physiological function of skeletal muscle after trauma. *Neural Regeneration Research* **2022**, *17*, 1617.
5. Guo, Y.; Min, Z.; Jiang, C.; Wang, W.; Yan, J.; Xu, P.; Xu, K.; Xu, J.; Sun, M.; Zhao, Y. Downregulation of HS6ST2 by miR-23b-3p enhances matrix degradation through p38 MAPK pathway in osteoarthritis. *Cell death & disease* **2018**, *9*, 1-15.
6. Tian, X.; Yang, H.; Fang, Q.; Quan, H.; Lu, H.; Wang, X. Circ_ZFR affects FABP7 expression to regulate breast cancer progression by acting as a sponge for miR-223-3p. *Thoracic Cancer* **2022**, *13*, 1369-1380.
7. Mawaribuchi, S.; Aiki, Y.; Ikeda, N.; Ito, Y. mRNA and miRNA expression profiles in an ectoderm-biased substate of human pluripotent stem cells. *Scientific reports* **2019**, *9*, 1-13.
8. Ye, J.; Luo, W.; Luo, L.; Zhai, L.; Huang, P. MicroRNA-671-5p inhibits cell proliferation, migration and invasion in non-small cell lung cancer by targeting MFAP3L. *Molecular Medicine Reports* **2022**, *25*, 1-8.
9. Thompson, E.M.; Stoker, A.W. A review of DUSP26: structure, regulation and relevance in human disease. *International Journal of Molecular Sciences* **2021**, *22*, 776.
10. Kefas, B.; Floyd, D.H.; Comeau, L.; Frisbee, A.; Dominguez, C.; Dipierro, C.G.; Guessous, F.; Abounader, R.; Purow, B. A miR-297/hypoxia/DGK-α axis regulating glioblastoma survival. *Neuro-oncology* **2013**, *15*, 1652-1663.
